# Stimuli-Responsive, Plasmonic Nanogel for Dual Delivery of Curcumin and Photothermal Therapy for Cancer Treatment

**DOI:** 10.3389/fchem.2020.602941

**Published:** 2021-01-20

**Authors:** Fadak Howaili, Ezgi Özliseli, Berrin Küçüktürkmen, Seyyede Mahboubeh Razavi, Majid Sadeghizadeh, Jessica M. Rosenholm

**Affiliations:** ^1^NanoBiotechnology Department, Faculty of Biological Science, Tarbiat Modares University, Tehran, Iran; ^2^Pharmaceutical Sciences Laboratory, Faculty of Science and Engineering, Åbo Akademi University, Turku, Finland; ^3^Department of Pharmaceutical Technology Faculty of Pharmacy, Ankara University, Ankara, Turkey; ^4^Polymer Reaction Engineering Department, Faculty of Chemical Engineering, Tarbiat Modares University, Tehran, Iran

**Keywords:** plasmonic nanogel, AuNP, curcumin, stimuli-responsive, photothermal therapy

## Abstract

Nanogels (Ng) are crosslinked polymer-based hydrogel nanoparticles considered to be next-generation drug delivery systems due to their superior properties, including high drug loading capacity, low toxicity, and stimuli responsiveness. In this study, dually thermo-pH-responsive plasmonic nanogel (AuNP@Ng) was synthesized by grafting poly (N-isopropyl acrylamide) (PNIPAM) to chitosan (CS) in the presence of a chemical crosslinker to serve as a drug carrier system. The nanogel was further incorporated with gold nanoparticles (AuNP) to provide simultaneous drug delivery and photothermal therapy (PTT). Curcumin's (Cur) low water solubility and low bioavailability are the biggest obstacles to effective use of curcumin for anticancer therapy, and these obstacles can be overcome by utilizing an efficient delivery system. Therefore, curcumin was chosen as a model drug to be loaded into the nanogel for enhancing the anticancer efficiency, and further, its therapeutic efficiency was enhanced by PTT of the formulated AuNP@Ng. Thorough characterization of Ng based on CS and PNIPAM was conducted to confirm successful synthesis. Furthermore, photothermal properties and swelling ratio of fabricated nanoparticles were evaluated. Morphology and size measurements of nanogel were determined by transmission electron microscopy (TEM), scanning electron microscopy (SEM) and energy-dispersive X-ray spectroscopy (EDX). Nanogel was found to have a hydrodynamic size of ~167 nm and exhibited sustained release of curcumin up to 72 h with dual thermo-pH responsive drug release behavior, as examined under different temperature and pH conditions. Cytocompatibility of plasmonic nanogel was evaluated on MDA-MB-231 human breast cancer and non-tumorigenic MCF 10A cell lines, and the findings indicated the nanogel formulation to be cytocompatible. Nanoparticle uptake studies showed high internalization of nanoparticles in cancer cells when compared with non-tumorigenic cells and confocal microscopy further demonstrated that AuNP@Ng were internalized into the MDA-MB-231 cancer cells via endosomal route. *In vitro* cytotoxicity studies revealed dose-dependent and time-dependent drug delivery of curcumin loaded AuNP@Ng/Cur. Furthermore, the developed nanoparticles showed an improved chemotherapy efficacy when irradiated with near-infrared (NIR) laser (808 nm) *in vitro*. This work revealed that synthesized plasmonic nanogel loaded with curcumin (AuNP@Ng/Cur) can act as stimuli-responsive nanocarriers, having potential for dual therapy i.e., delivery of hydrophobic drug and photothermal therapy.

## Introduction

Breast cancer is the second most common cause of cancer among women worldwide (Bray et al., 2018). Breast cancer treatment usually requires a combination of chemotherapy, radiotherapy, and surgery (Waks and Winer, 2019). However, conventional chemotherapy agents suffer from a lack of aqueous solubility, lack of selectivity, and are subject to multidrug resistance. Nanotherapeutics are rapidly developing to overcome the limitations of traditional drug delivery systems (Dong et al., 2019; He et al., 2020; Liu et al., 2020). Common anticancer drugs damage peripheral tissues and cells other than the targeted tissue, and here, using herbal medicines could be a safer alternative choice to avoid harmful side effects (Pavan et al., 2016; Unlu et al., 2016; Oun et al., 2018). Curcumin is the main component of turmeric, a polyphenol with low molecular weight. *In vivo* studies have shown that curcumin helps to prevent metastatic progression in models of breast cancer and to inhibit cancer cell proliferation and invasion by downregulating the PI3K/Akt signaling pathway (Xu et al., 2014). However, the most significant problems restricting its use in therapy are the poor solubility, instability, rapid metabolism, systemic elimination, and inadequate tissue absorption of curcumin (Kunnumakkara et al., 2017). For instance, the administration of 8 grams of curcumin per day led to only 1.77 μM absorption in the body (Unlu et al., 2016). Thus far, various approaches have been used to overcome curcumin-related issues, some of which are using nanocarriers such as phospholipids, micelles, liposomes, and polymeric nanoparticles (Gatti and Perucca, 1994; Kunwar et al., 2006; Li et al., 2007; Ma et al., 2007; Suresh and Srinivasan, 2007; Takahashi et al., 2009; Bani et al., 2016; Esmatabadi et al., 2018; Farsani et al., 2020). Nanogels are water-soluble crosslinked hydrogel materials that have both hydrogel and nanoparticle properties at the same time, in addition to controlled drug release capability. These carriers thus provide a polymeric nanotechnological approach which has exceptional characteristics such as high drug loading capacity, high stability, responsiveness to a wide variety of environmental stimuli whereby they may shrink or swell in response to pH or temperature change, resulting in the release of the drug under specific conditions. Hence, various bioactive compounds can be encapsulated into nanogels, exceptionally also hydrophobic drugs (Maya et al., 2013). In this context, the use of a responsive polymer for the synthesis of the nanogel is one method of delivering drugs to cancer cells. PNIPAM is a pH and temperature-responsive polymer that can be synthesized via free-radical polymerization. It can be effectively functionalized, making it useful in a range of medical applications. It undergoes a reversible lower critical solution temperature (LCST) around 32°C in water, in which the phase transition from a swollen hydrated state to a shrunken dehydrated state takes place; losing about 90% of its volume(Ormategui et al., 2012). Since PNIPAM expels its liquid content at temperatures close to the human body, several researchers have investigated its potential applications in tissue engineering, biosensors and controlled drug delivery, but its use is limited due to its synthetic nature (Guan and Zhang, 2011). In comparison, the use of chitosan, a natural polymer, enhances the biodegradability and biocompatibility of the synthesized nanogel and improves PNIPAM's LCST by rising it to a drug-release-appropriate temperature at or above body temperature (Oh et al., 2010; Pereira et al., 2015).

**Figure 1:**
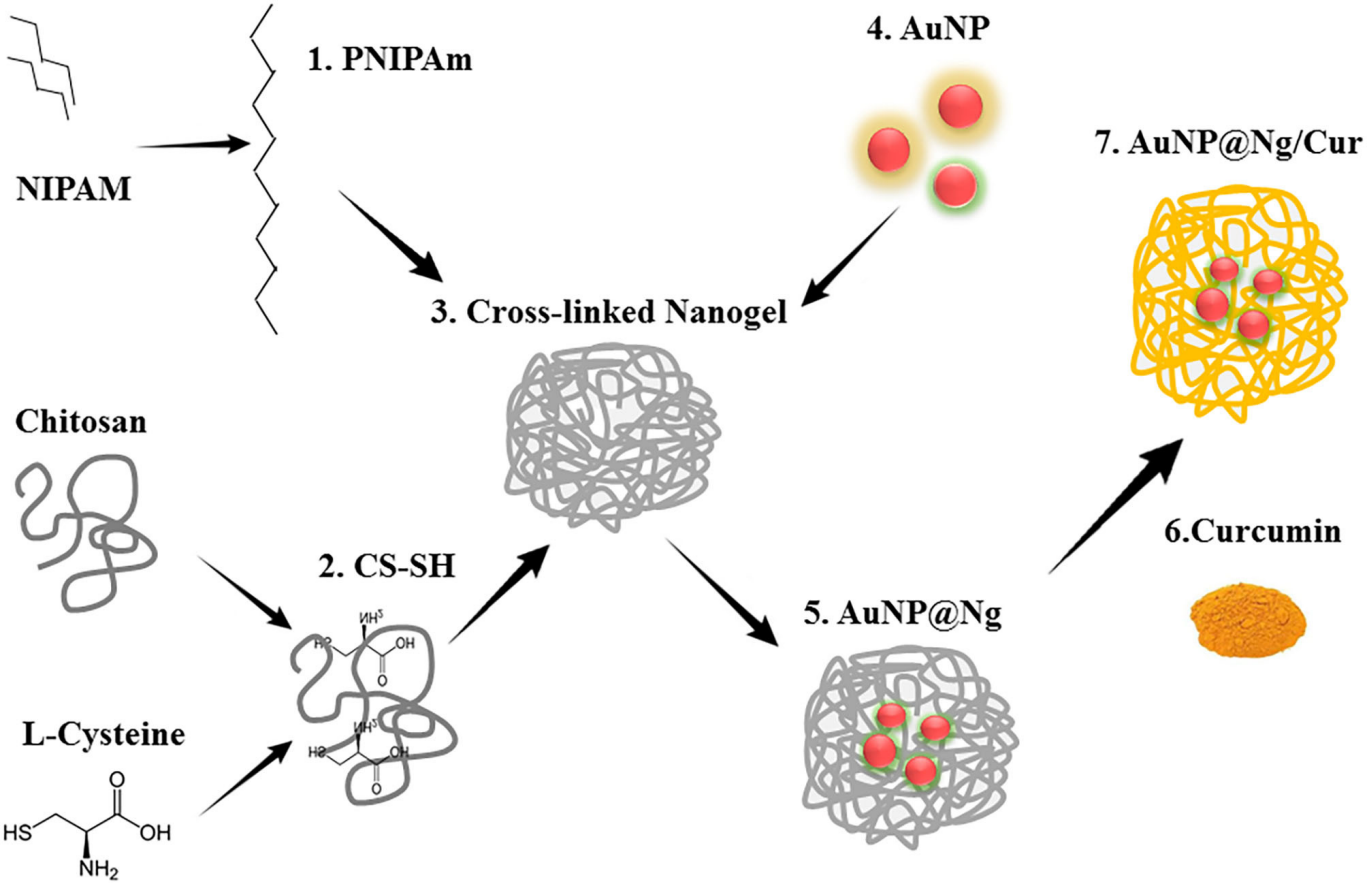
Graphical abstract showing the steps of formation of AuNP@Ng/Cur. Chitosan was thiolated using L-Cysteine, afterwards, it crosslinked to PNIPAM to form the nanogel. The next step was the incorporation of AuNP to nanogel via semi covalent bond of the gold nanoparticle to SH groups in the nanogel. In the end, using the incubation method, the curcumin was loaded to AuNP@Ng.

AuNP has attracted tremendous as promising PTT agents for the treatment of malignant tumors with impressive localized surface-plasma-resonance (LSPR) properties including absorption in the NIR region, which is essentially required for PTT. Additionally, AuNP is visible in confocal laser scanning microscopy through internal reflection, which provides a tool for tracking the uptake of nanoparticles by cells (Shukla et al., 2005; Murphy et al., 2008; Kim et al., 2015). The present contribution reports the development of stimuli-responsive plasmonic nanogel for combinable curcumin and photothermal therapy, composed of AuNP and PNIPAM-chitosan nanogel. In this work, citrate capped AuNP were synthesized using the Turkevich method and were incorporated into the nanogel in order to introduce imaging and PTT capabilities to the synthesized nanogels (Figure 1). Physicochemical properties of developed nanogel including morphology, size, net surface charge (zeta potential), swelling ratio, thermal analysis and size dispersity were characterized. Developed nanogel delivery system not only showed excellent cytocompatibility but could also be utilized as a cellular imaging probe due to the strong light scattering property of incorporated AuNP. Moreover, the chemotherapy, laser-induced PTT therapy efficacy and quantitative cellular uptake of multifunctional AuNP@ Ng were compared by using curcumin as a chemotherapeutic agent and testing single and combined therapy on MDA-MB-231 breast cancer cells and non-tumor cell line MCF 10A. The results showed sustained drug release from nanoparticles and a significant synergistic effect upon combined therapy.

## Experimental

### Materials

The medium molecular weight chitosan (CS) (190–310 kDa and degree of deacetylation of 75–85%), L-cysteine 97%, 1-ethyl-3-(3-dimethylaminopropyl)carbodiimide(EDC) 99%, N-isopropylacrylamide 97%, acetic acid glacial ≥99%, N, N′-methylenebis(acrylamide) (MBA)99%, ammonium persulfate (APS) 98%, hydrochloric acid 37%, gold(III) chloride trihydrate ≥99.9%, sodium citrate dihydrate ≥99%, curcumin powder from Curcuma longa (Turmeric), crystal Violet solution 2.3% (w/v), paraformaldehyde 95% (PFA), hydrocortisone, cholera toxin and insulin all were commercially available and supplied from Sigma-Aldrich Co. Dulbecco's Modified Eagle's Medium (DMEM), Penicillin-Streptomycin, Dulbecco's Phosphate Buffered Saline, Penicillin-Streptomycin(pen/strep) and L-Glutamine were obtained from Lonza. Dil Stain (1,1'-Dioctadecyl-3,3,3',3'-Tetramethylindocarbocyanine Perchlorate ['DiI'; DiIC18(3)], Gibco fetal bovine serum (FBS), Gibco DMEM/F12–Dulbecco's Modified Eagle Medium: Nutrient Mixture F-12, Gibco horse serum and MEM Non-Essential Amino Acids Solution (100X) (NEAA) were purchased from Thermofisher. The epidermal growth factor was purchased from Abcam. AlamarBlue Cell Viability Reagent was obtained from TCI Europe and Vectashield DAPI mounting media from Vector Labs.

### Synthesis of AuNP@Ng/Cur

The chitosan was thiolated via attaching L-cysteine to chitosan by the formation of amide bonds to connect the AuNP with Ng, the degree of thiol group modification was measured spectrophotometrically with Ellman's reagent (Schmitz et al., 2008). Subsequently, thiolated Chitosan-PNIPAM Ng were synthesized employing the free radical surfactant-free emulsion polymerization method following the previously described procedure with some desired modification. Briefly, to ionize free -NH_2_ group of CS, 500 mg of thiolated CS (4.618 ± 0.25 μmol SH /mg CS) was dissolved in 20 ml acetic acid (1%) solution in a 250 ml three-neck round bottom flask on the heater stirrer under nitrogen atmosphere. After the absolute dissolution of ionized chitosan, 500 mg of NIPAM dissolved in 80 ml of Milli-Q water and 1.7 mg MBA as a crosslinker were added to the reaction, and the temperature was increased gradually to the 80°C. The polymerization was initiated by adding 0.85 ml APS (0.05M in water) as an initiator to the flask after purging with nitrogen for about 30–40 min. The reaction was proceeded for 3 h at 80°C and then terminated by cooling down to room temperature. The obtained samples were filtered with a 1 μm membrane filter and then dialyzed with a dialysis membrane (12,000 Da MWCO) against Milli-Q water, the water was changed periodically 15 times for 3 days. For long-term storage, nanogel was freeze-dried overnight (Echeverria et al., 2015; Khan et al., 2015).

The Turkevich method was used to synthesize Citrate capped AuNP (Kimling et al., 2006). Subsequently, in order to attach AuNP to nanogel via semi covalent interaction of SH group of Ng and AuNP, 1 ml (5 mg/ml) of synthesized nanogel was added to 5 ml (1 mg/ml) of AuNP suspension and continuously stirred for 2 days at room temperature in the dark. The content was then dialyzed against Milli-Q water overnight using a dialysis membrane (12,000 Da MWCO), and the water was changed at specified intervals (Ding et al., 2009). Curcumin was loaded to the nanogel using the incubation method. Briefly, curcumin (0.25 and 0.5 mg/ml) was dissolved in ethanol with 1 mM concentration and added to the 1 ml AuNP@ nanogel and 1 ml nanogel solution dropwise and at a temperature higher than 32°C under constant stirring in the dark for 24 h. Finally, nanogel was centrifuged by Thermo Scientific™ Sorvall LYNX 4000 Centrifuge at 8,000 rpm for 20 min and lyophilized overnight in Heto CT60e freeze-dryer for storage (Luckanagul et al., 2018).

### Characterization of AuNP@Ng/Cur

To analyze the structure of synthesized nanogel, the FTIR spectra of CS, thiolated CS, NIPAM, SH- CS-PNIPAM were obtained using Perkin-Elmer Spectrum Two, scanning from 4,000 to 400 cm^−1^.

In order to calculate the deswelling ratio of synthesized nanoparticles, the average hydrodynamic size of nanogel and AuNP@Ng was determined at different temperatures and then the deswelling ratio was calculated according to the following equation (Zhao et al., 2019; Agnihotri et al., 2020):

(1) $$\begin{array}{r} Deswelling\;ratio=S_{s}/S_{d} \end{array}$$

Where the S_s_ is the size of Ng in room temperature and S_d_ is the size of Ng above LSCT which we measured at 25, 32, 37, 42, and 60°C temperature.

Moreover, polydispersity index (PDI) and zeta potential of nanogel were determined at 25, 32, 37, 42, and 60°C by a Malvern Zeta Sizer ZS (PCS, Malvern Instruments Ltd). The optical properties of AuNP@Ng were obtained by Themo scientific 2000c UV-Vis spectrophotometer using a 1-cm-wide quartz cuvette. TEM was conducted using JEM-1400 Plus TEM following negative staining with uranyl acetate to confirm the size of nanogel as well as the morphology. TEM images were illustrated using Fiji Image J software.

Using a field-emission SEM (FESEM) TeScan-Mira III model, 10 times diluted AuNP@Ng/Cur nanoparticles were sputter-coated with gold after dropping it on the grid to achieve more homogeneous and transparent images. SEM and Elemental analysis for AuNP@Ng/Cur was acquired by EDX X-ray detector of SEM (Thermo Scientific LEO Gemini 1530 model (using uncoated sample.

The thermal analysis of synthesized AuNP@Ng/Cur, AuNP, and control (nanogel) were characterized by measuring the temperature at different time points of 0, 3, 5, 7, and 10 at two different concentrations of 2.5 and 5 μg/ml under NIR exposure(Fu et al., 2018). Using thermal camera GUIDE B from Sensmart, the thermal image and measured temperature were obtained and the temperature change (ΔT) was determined using the following equation (Fu et al., 2018):

(2) $$\begin{array}{r} \Delta T=T_{2}-T_{1} \end{array}$$

ΔT is the temperature difference between two heating times. T_2_ is the temperature measured for one nanoparticle at one-time point and T_1_ is the temperature measured for control at the same time point.

### Encapsulation Efficiency (EE%) and Drug Loading (DL%)

The EE% of AuNP@Ng/Cur was determined by measuring the amount of curcumin indirectly from the supernatant. Briefly, Curcumin (0.25 and 0.5 mg/ml) was dissolved in ethanol and then added to both 1 ml Ng and 1 ml AuNP@Ng and stirred for 24 h at room temperature by protecting from light. The following day, nanogel was centrifuged at 3,000 rpm for 5 min. The supernatant was collected, and the amount of excessive curcumin was estimated by using Thermo scientific 2000c UV-Vis spectrophotometer at 450 nm. The EE% and gDL% were calculated using the following equations (Equations 3, 4) (Sarika et al., 2016; Luckanagul et al., 2018).

(3) $$\begin{array}{r} EE\%=\frac{Total\;amount\;of\;feeding\;curcumin-free\;curcumin}{Total\;amount\;of\;curcumin}*100 \end{array}$$

(4) $$\begin{array}{r} DL\%=\frac{Total\;amount\;of\;feeding\;curcumin\;-Free\;curcumin}{Weight\;of\;nanogel\;}*100 \end{array}$$

### *In vitro* Curcumin Release

*In vitro* curcumin release from nanogel and AuNP@Ng was carried out in a shaker incubator in phosphate-buffered saline containing Tween 80 (0.5% w/v) in two different pH values (pH 7.4 and pH 5.5) and two different temperatures (25 and 37°C) (*n* = 3) for 72 h. Samples were collected (1 ml) at defined time intervals and centrifuged at 13,000 rpm for 8 min. The absorption intensity of the diluted supernatant in DMSO was measured at 450 nm(Sarika and Nirmala, 2016).

### Cell Studies

#### Cell Culture and Maintenance

Human triple-negative breast cancer cell line MDA-MB-231 was cultured with Dulbecco's modified Eagle's medium (DMEM) supplemented with 10% heat-inactivated fetal bovine serum (FBS), 2 mM L-glutamine, 0.1 mM MEM Non-Essential Amino Acids (NEAA), 100 IU/ml penicillin and 100 ug/ml of Streptomycin at 37°C with 5% CO_2_. Cells were passaged when they reached 80–90% confluency. Human breast epithelial cell line MCF10A was cultured with Dulbecco's Modified Eagle Medium: Nutrient Mixture F-12 (DMEM/F12) supplemented with 5% heat-inactivated horse serum, 100 IU/ml penicillin and 100 ug/ml of Streptomycin, 20 ng/ml Epidermal Growth Factor, 0.5 mg/ml hydrocortisone, 10 μg/ml insulin and 100 ng/ml cholera toxin.

#### Cytotoxicity and Chemo-Photothermal Efficacy of AuNP@Ng/Cur Nanoparticles

Alamar Blue cell proliferation assay was carried out to investigate the cytocompatibility of AuNP@Ng and AuNP@Ng/Cur nanoparticles. Briefly, MDA-MB-231 cells (5 × 10^3^ cells/cm^2^) and MCF10A cells (7.5 × 10^3^ cells/cm^2^) were cultured in a 96-well plate overnight and cell media was replaced with pre-warmed fresh media containing AuNP@Ng (30-60-120 μg/ml) with the corresponding concentrations of AuNP(5-10-20 μg/ml), and nanogel (25-50-100 μg/ml) individually (*n* = 4). Cytotoxicity of nanoparticles was evaluated after 24 and 48 h incubation. Subsequently, the Alamar Blue reagent was added to each well (10% final concentration) as suggested by the manufacturer and incubated at 37°C for 4 h to allow resazurin to undergo metabolic reaction. Fluorescence intensity of reduced form of resazurin was measured spectrophotometrically at 570 nm excitation and 580–600 nm as emission wavelength (Wu et al., 2011; Osterman et al., 2016; Tang et al., 2020) by using Thermo Scientific Varioskan Flash multi-plate reader. The percentage of cell proliferation was reported relative to untreated cells (100% viability) according the following equation using relative fluorescence units (RFU) (Eilenberger et al., 2018):

(5) $$\begin{array}{r} Cell\;viability\;\%=\frac{Experimental\;RFU\;with\;chemical\;compound-background\;RFU\;}{Untreated\;cell\;control\;RFU\;value-background\;RFU\;}*100 \end{array}$$

The data in each time point were normalized by their corresponding control. A similar procedure was followed to determine the cytotoxicity of AuNP@Ng/Cur. Cells were incubated with the cell media suspension of free curcumin, AuNP@Ng and AuNP@Ng/Cur containing 10, 25, 50, and 100 μg/ml curcumin for 24 and 48 h (*n* = 4). At designated time intervals, Alamar Blue reagent was added to suspension, and fluorescence was measured after 4 h incubation. The autofluorescence of curcumin was subtracted from the acquired measurements.

For photothermal and curcumin-photothermal treatment, the MDA-MB-231 cells were incubated with AuNP, AuNP@Ng, AuNP@Ng/Cur containing 5 μg/ml and 2.5 μg/ml AuNP for 24 h. Following day, the MDA-MB-231 cells were exposed to 2.19 W/cm2, 808 nm NIR laser for 10 min by an ~6 mm focused spot size, and cell viability was determined by Alamar Blue assay described as previously (Yang et al., 2017; Ong et al., 2019; Park et al., 2019). In parallel, cells were stained with crystal violet after NIR laser treatment to demonstrate the effect of chemo-photothermal treatment on MDA-MB-231 cells visually. Cell media was discarded, and the cells were washed 2 times with 300 μl of PBS, following with the fixation using 200 μl of 4% PFA for 7 min at RT. Finally, the fixed cells were stained with 200 μl crystal violet stain (0.1% in 20% methanol) for 5 min and subsequently washed twice with 300 μl Milli-Q water to remove the excess dye and left to dry. Cell images were acquired by Thermo Fisher EVOS XL Core Cell Imaging System (Palmieri et al., 2015).

#### Cellular Uptake Study of AuNP and AuNP@Ng by Flow Cytometry and Confocal Microscopy

Cellular internalization of AuNP, AuNP@Ng, and Ng was investigated for cancer cell line MDA-MB-231 and non-tumorigenic cell line MCF10A by using flow cytometry. In brief, MDA-MB-231 and MCF10A cells were seeded in the 12-well plates for overnight attachment with the concentration of (60 × 10^3^ cells/cm^2^) and (90 × 10^3^ cells/cm^2^), respectively. One day later, the cell media was substituted by fresh media containing AuNP (20 μg/ml), nanogel (100 μg/ml), and AuNP@Ng (120 μg/ml) and incubated at 37°C, 5% CO_2_ for 24 h (*n* = 3). Afterwards, cells were harvested with trypsinization, washed twice with ice cold PBS and resuspended in 250 μl of PBS, kept on ice until analysis (Bansal et al., 2020). Mean fluorescence intensity was recorded by using a 561 nm excitation laser and the emission was collected by using RFP 582/15 band pass filter with LSRFortessa (BD Sciences, San Diego, CA, USA). The analyzer was set to record 10,000 events per sample. Flowing Software (Open source software, Turku Center for Biotechnology, Finland) was used for data analysis and WinList 9.0 was used for the visualization of the overlay histograms.

For confocal microscopy, MDA-MB-231 cells were seeded on autoclaved coverslips (19ø) at the density of 16 × 10^3^ cells/cm^2^ in 12-well cell culture plates to examine the cellular uptake of AuNP@Ng and Au@Ng/Cur and incubated for 24 h at 37°C. Following day, cells were treated with AuNP, nanogel, AuNP@Ng, and AuNP@Ng/Cur with the concentration range of 2.5–5 μg/ml AuNP, by replacing fresh media containing nanoparticles. After 24 h incubation, cells were rinsed with PBS, and the cell membrane was stained by incubating cells with 1 ml fresh cell media containing 1 μg/ml of DiI hydrophobic dye for 10 min at 37°C. Stained cells were fixed with 4% PFA for 10 min at room temperature, washed thrice with PBS followed by a final washing with MiliQ water. Coverslips were mounted with VECTASHIELD mounting medium and cellular uptake of nanoparticles was imaged by Leica TCS SP5 confocal microscopy. AuNP were detected by reflection imaging by using 488 nm excitation, 481–493 nm emission, and DiI cell membrane dye was detected with 561 nm excitation, 563–612 nm emission wavelength. Images were illustrated using Fiji Image J software (Liu et al., 2015; Senthilkumar et al., 2015).

### Statistical Analysis

Statistical analysis was carried out using two-way ANOVA accompanied by Tukey's multiple comparisons test and two-tailed Student's *t*-test in GraphPad Prism 6 software. The collected data is presented in terms of “mean ± standard deviation” values. If the *p* < 0.05, differences are considered statistically significant.

## Results and Discussion

### Synthesis and Characterization of Nanogel, AuNP@Ng and AuNP @Ng/Cur

In this study, thermo-responsive plasmonic nanogel was synthesized in order to increase the solubility of curcumin and prevent its rapid degradation metabolism. AuNP were incorporated into the nanogel to add PTT features to the product by thiolating the chitosan using L-cysteine, and the amount of free thiol group on chitosan was quantified using Ellman's reagent. As shown in Table 1, by increasing the EDC amount from 150 to 200 mM, the ratio of thiol group on the CS backbone increased from 4.618 ± 0.25 to 9.247 ± 0.381 μmol/mg. Afterward, NIPAM was grafted to thiolated chitosan and crosslinked using MBA. NIPAM/MBA was polymerized by a free radical surfactant-free emulsion polymerization method to form CS-based Ng. For this purpose, PNIPAM and MBA were grafted to CS by the formation of an amide bond between the free -NH_2_ group of chitosan and the carboxyl group of NIPAM as well as by free-radical generated onto the CS (Wu et al., 2018). Polymer networks have been formed using MBA as a crosslinker during polymerization. AuNP were then synthesized by the Turkevich process, and incorporated by the semi-covalent bond of -SH groups in the thiolated CS into the nanogel. Finally, curcumin was loaded at a temperature higher than LCST using the incubation method to facilitate the hydrophobic interaction between curcumin and nanogel, which can be clarified based on the phase change from hydrophilic to hydrophilic PNIPAM (Asghar et al., 2017; García-Peñas et al., 2019).

**Table 1:** Characterization of thiolated CS using Ellman's reagent.

**CS-SH**	**CS solution (12.5 mg/ml)**	**EDC (mM)**	**L-cystiene(mg)**	**Total thiol groups (μmol/mg CS) ± SD**	**Free thiol group (umol/mg) ± SD**
1	0.5 ml	150	62	4.618 ± 0.25	0.967 ± 0.013
2	0.5 ml	200	62	9.247 ± 0.381	2.289 ± 0.062

**Figure 2:**
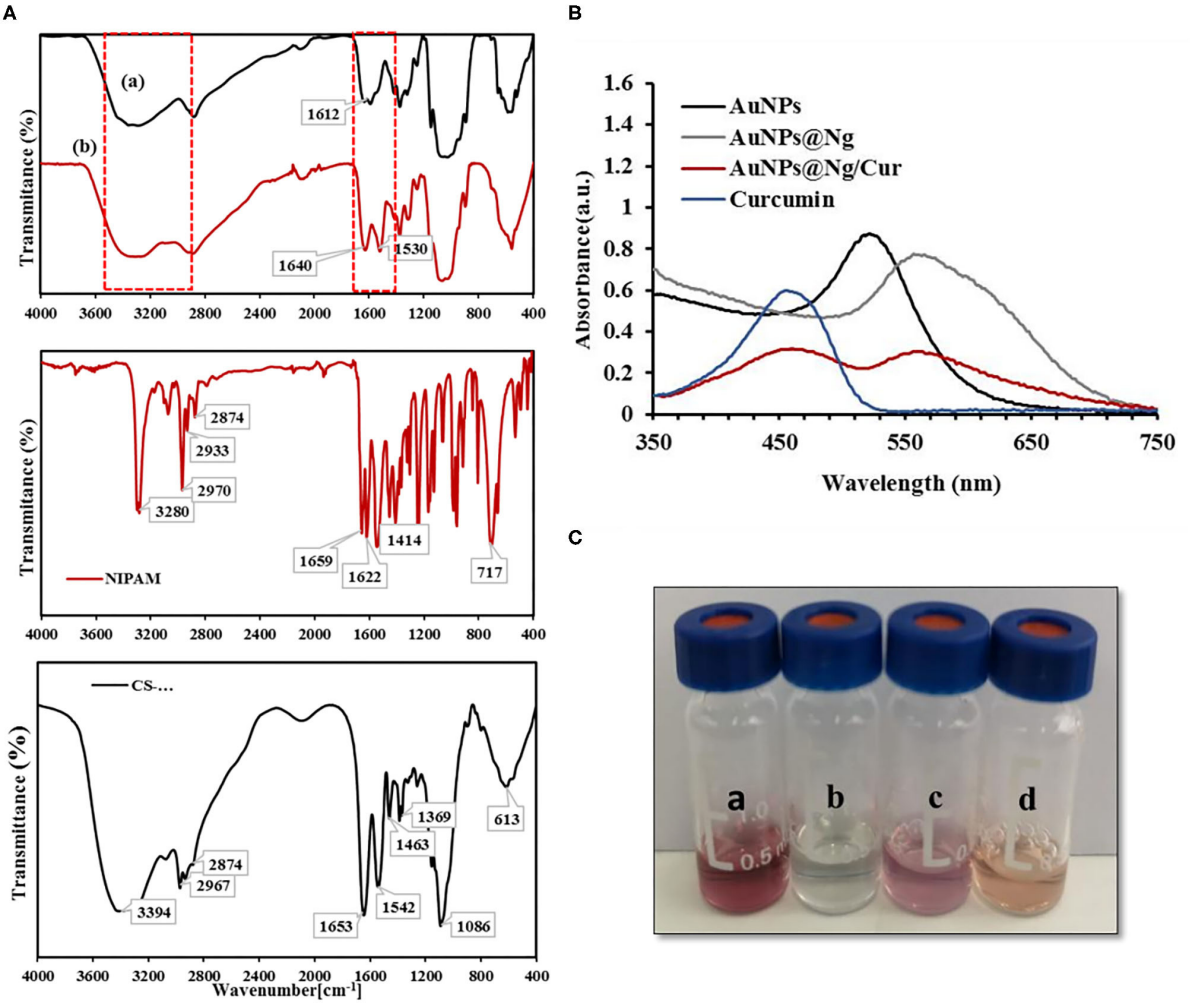
FT-IR spectra of chitosan **(a)**, thiolated chitosan **(b)**, pure NIPAM and Ng. **(A)** UV-VIS spectrum of AuNP, Curcumin, AuNP@Ng, and AuNP@Ng/Cur **(B)**. Physical appearance of AuNP **(a)**, Ng **(b)**, AuNP@Ng **(c)**, and AuNP@Ng/Cur **(d) (C)**.

FTIR spectroscopy was used to demonstrate the presence of different functional groups in the nanogel system. The FTIR spectra of pure NIPAM, chitosan, thiolated chitosan, and nanogel are demonstrated in Figure 2A, The spectrum of thiolated-CS showed deformation of the –NH- stretching signal at 3225 cm^1^ compared to chitosan spectra [Figure 2A(a,b)], which confirm the formation of (C-NH) amide bond by attachment of amine groups of chitosan to carboxyl groups of L-cysteine. Moreover, the amine groups of chitosan have sharp peaks in the wavelength between 1,450 and 1,650, and these peaks get weaker after thiolation with the L-cysteine as a result of the formation of amide bonds (Esquivel et al., 2015). In NIPAM spectrum, peaks around 3,280, 1,622, and 1,414 cm^−1^ are assigned to C-H, C=C and CH_2_ = respectively, characteristic of vinyl monomer (Figure 2A). Compared to NIPAM and thiolated chitosan spectra, the CS-PNIPAM spectrum showed some new signals and many others either disappeared or deformed. As shown in Figure 2A, the FTIR spectrum of pure NIPAM has peaks characterizing double bonds, but the FTIR spectrum of nanogel did not show any characteristic peak of the double bond in the range of 1,600–1,650 cm^−1^ (C=C aliphatic and aromatic) and 610–990 cm^−1^ (stretching mode of vinyl double bonds). Similarly, no characteristic peaks of cis-trans and substituted groups with double bonds (700–900 cm^−1^) or a peak for =C-H could be found. Furthermore, the broad and intense peak near 3,292 cm^−1^ suggests N-H stretching in the spectrum of Ng. This peak showed hydrogen bonding due to the presence of water of hydration attached to the polymer and confirmed gel formation. Thus, our FTIR spectrum results suggest successful CS-PNIPAM polymerization and nanogel formation (Figure 2A; Kim et al., 2005; Shah et al., 2013; Esquivel et al., 2015; Khan et al., 2015).

The interaction of AuNP [Figure 2C(a)] with Ng [Figure 2C(b)] and curcumin loading in AuNP@Ng [Figure 2C(d)] was further investigated with UV-spectroscopy. As shown in Figure 2B(a) peak at 522 nm can be ascribed to the plasmon resonance effect of the AuNP. After the interaction of AuNP with nanogel, the AuNP peak had a lower absorbance value and showed a redshift in wavelength; this shift in the plasmon resonance effect of the AuNP confirms the interaction of AuNP with Ng and formation of AuNP@Ng (Park et al., 2018). The UV-VIS spectroscopy was also employed to confirm the curcumin loading of AuNP@Ng. As shown in Figure 2B, there is a peak at 450 nm in addition to the AuNP peak in the spectrum of AuNP@Ng/Cur, which indicated the encapsulation of curcumin by the nanogel. The difference in the intensity of peaks is due to the difference in concentration of AuNP and curcumin in AuNP@Ng and AuNP@Ng/Cur samples compared to bare curcumin and AuNP (Alam et al., 2012).

**Figure 3:**
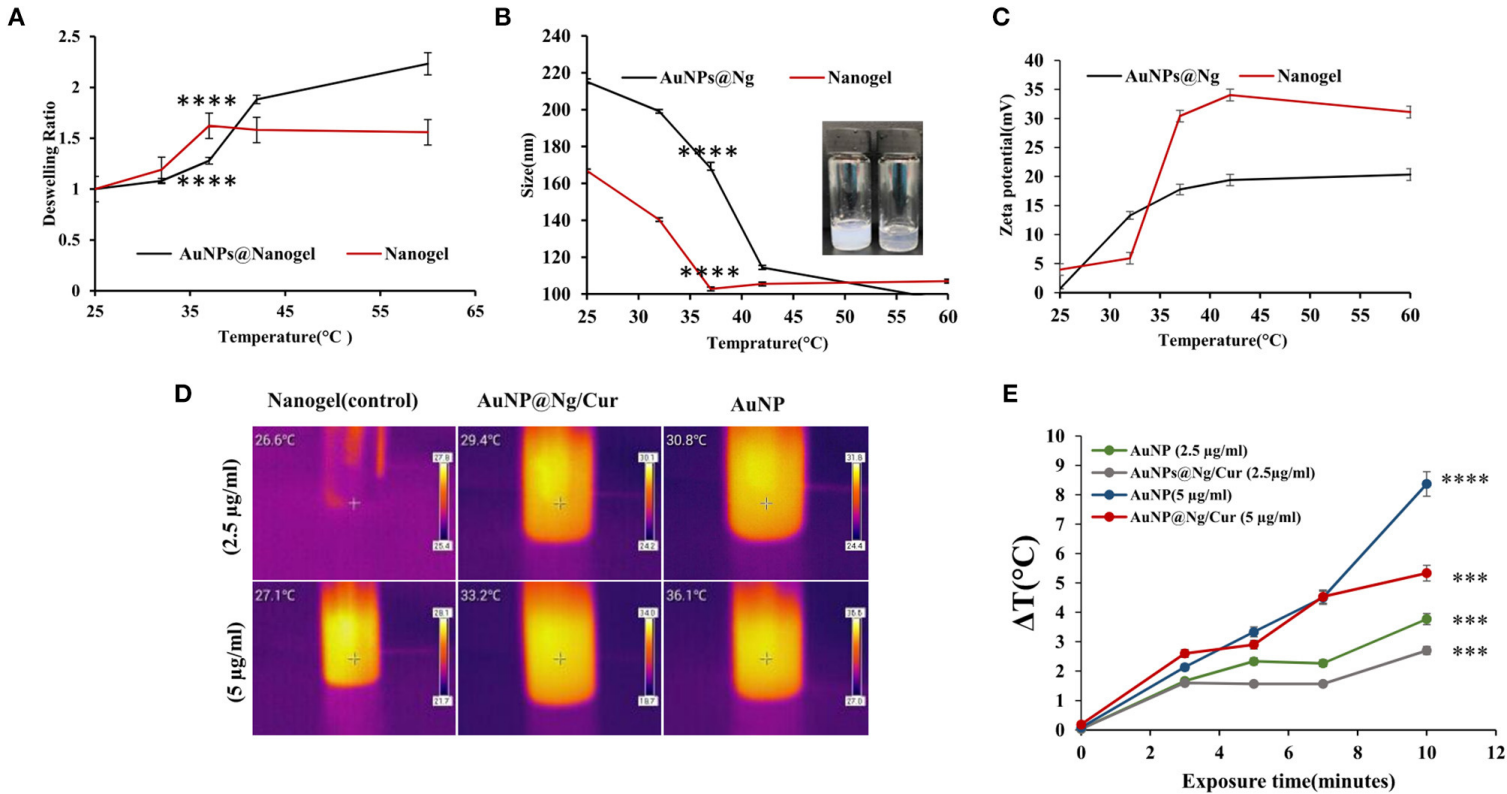
Deswelling ratio of Nanogel and AuNP@Ng at different temperatures. **(A)** The average sizes of the Ng and AuNP@Ng at different temperatures (25–60°C) measured by DLS, physical appearance of Ng at different temperatures of 25 and 42°C. **(B)** Zeta potential of AuNP@Ng and Ng at different temperatures. **(C)** Thermal images of Ng, AuNP and AuNP@Ng/Cur in two different concentrations after 10 min exposure of NIR irradiation. **(D)** ΔT(°C) of AuNP and AuNP@Ng/Cur solutions at different time points after NIR irradiation. **(E)** Two-way ANOVA followed by Tukey's multiple comparisons test was performed to investigate the significant difference (****p < 0.0001; ***p < 0.0001–0.001).

Formulated AuNP@Ng were characterized in terms of dynamic light scattering (DLS) analysis, to determine the average hydrodynamic diameter, polydispersity index (PDI), and zeta potential (ZP). The hydrodynamic diameter, PDI, and ZP of bare AuNP were 22.24 nm, 0.305, and −31.1 mV, respectively. Besides, DLS results showed a larger average particle size for AuNP@Ng (215.16 ± 5.78 nm) compared to the bare nanogel (166.8 ± 0.60 nm) (Table 2). The results could be explained with the size of nanogel depending on how many AuNP could attach to the CS-SH in the cross-linked nanogel, resulting in a reduction of monodispersity. The size of plasmonic nanogel after curcumin loading was found to be 226 ± 1.49 nm, and it was seen that curcumin loading did not significantly change the nanoparticle size. This result may be due to the high loading capacity of the plasmonic nanogel attributed to its softness and flexibility, where curcumin can be trapped easily (Karg et al., 2019). Changes in the size and swelling ratio of nanoparticles under different environmental conditions may have an impact on the controlled release of drugs. Therefore, to observe the thermal response behavior of synthesized nanoparticles, DLS measurements were applied. For this reason, the size of nanogel and AuNP@Ng was measured at different temperatures: 25, 32, 37, 42, and 60°C and the deswelling ratio was calculated according to Equation 1. As shown in Figure 3B, by increasing the temperature to 37°C the size of AuNP@Ng and nanogel were significantly reduced (*P* < 0.0001), and this reduction continued slightly above 37°C. Accordingly, as shown in Figure 3A, the deswelling ratio significantly increased (*P* < 0.0001). This change in size demonstrated the thermo-responsive behavior of synthesized nanogel due to the low critical solution temperature property of PNIPAM, which makes the nanogel collapse in temperatures higher than LCST. The LCST of PNIPAM is 32°C, but the use of chitosan increased the LCST of PNIPAm to 37°C, making the drug delivery system more suitable for drug release at body temperature (Ashraf et al., 2016). Thermally-induced color change from transparent to milky was observed by increasing the temperature of Ng as a result of phase transition from swollen state to shrunk state (Figure 3B; Christau et al., 2016).

**Table 2:** Average size, PDI, and Zeta potential of AuNP, Ng, AuNP@Ng, and AuNP@Ng/Cur determined using DLS and TEM.

**Nanoparticle**	**TEM size (nm)**	**DLS size (nm)**	**PDI**	**Zeta potential (mV)**
AuNP	19.57 ± 1.22	22.24 ± 0.131	0.051	−31.1
Ng	167.81 ± 4.74	166.8 ± 0.60	0.195	3.97
AuNP@Ng	197.94 ± 2.33	215.16 ± 5.78	0.228	0.655
AuNP @Ng/Cur	214.77 ± 4.28	226 ± 1.49	0.354	0.676

Zeta potential is one of the most crucial parameters in colloidal systems that affect the fate of nanoparticles in drug delivery, cellular uptake, and drug interactions with the surrounding environment (Honary and Zahir, 2013). In this study, the zeta potential measurement of AuNP@Ng at different temperatures showed a considerable increase from 0.665 mV at 25°C to 17.76 ± 0.64 mV at 37°C, still gradually increasing to 20.33 ± 0.305 mV at 60°C. Nanogel has a more abrupt change in zeta potential compared to AuNP@Ng at 37°C. This data supports the shrinkage of the nanogel by increasing the temperature, which is due to the placement of positively charged isopropyl groups of polymer on the surface of nanogel in temperatures higher than LCST (Figure 3C; Utashiro et al., 2017).

The presence of AuNP incorporated in the nanogel improves the therapeutic effect of the formulation by means of AuNP's surface plasmon resonance property generating heat after exposure to NIR irradiation. In order to demonstrate the photothermal activity of synthesized nanoparticles, nanogel, AuNP, and AuNP@Ng/Cur thermal analysis after exposure of 808 nm NIR laser were assessed at different time points. According to statistical analysis, calculated ΔT according to Equation 1 at different time points of 0, 3, 5, 7, and 10 min showed significant time and dose dependent differences in comparison with the control samples (*P* < 0.001). Both AuNP@Ng/Cur and AuNP samples, respectively, exhibited 5.1 and 8.4°C temperature raise compared with control (Figure 3E; Fu et al., 2018). The thermal images show the temperature change of samples after 10 min of exposure of NIR radiation for two different concentration of AuNP, nanogel, and AuNP@Ng/Cur (Figure 3D).

**Figure 4:**
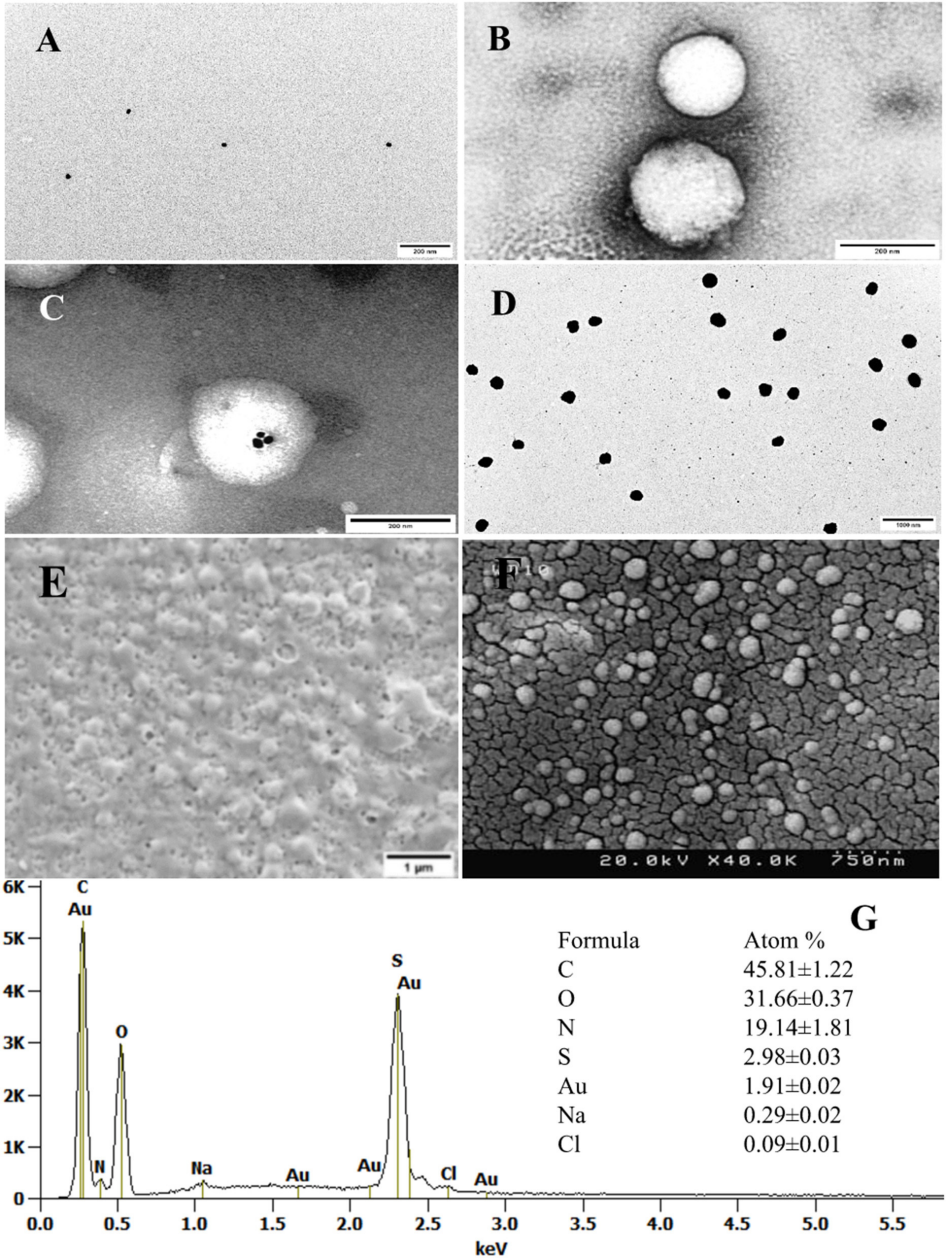
Electron micrograph and analysis. TEM images of the AuNP **(A)**, Negatively stained Ng **(B)**, and AuNP@ Ng with uranyl acetate (scale bar 200 nm) **(C)** AuNP@ Ng loaded with Curcumin without being negatively stained (scale bar 1000 nm) **(D)**. Untreated SEM image of AuNP@Ng/Cur (scale bar 1μm) **(E)**. Gold sputter-coated AuNP@Ng/Cur FESEM image (scale bar 750 nm) **(F)**. EDX spectrum and quantitative atom% information of AuNP@Ng/Cur **(G)**.

According to TEM analysis, AuNP (Figure 4A), nanogel (Figure 4B), AuNP@Ng (Figure 4C), and AuNP@Ng/Cur (Figure 4D) were found to be of spherical shape, showing an excellent monodispersity with a corresponding hydrodynamic size of 19.57 ± 1.22, 167.81 ± 4.74, 197.94 ± 2.33, and 214.77 ± 4.28 nm, respectively (Table 2). The hydrodynamic size of the nanoparticles obtained by TEM analysis was found smaller compared to the diameter obtained by the DLS method, which is expected due to the difference in the operating principles of these two measurement methods (Kaasalainen et al., 2017). Attributed to the impact of the dispersant on the hydrodynamic diameter of the nanogel, it is predicted that the values obtained from DLS would be slightly higher than TEM, which reveals the solid state size of Ng upon TEM imaging (Zhao et al., 2011).

Due to the impact of drying on the nanogel network structure, FESEM and SEM imaging showed distributed spherical AuNP@Ng/Cur with some heterogeneous nanoparticles (Figures 4E,F) (Sidhu et al., 2019). The AuNP@Ng/Cur elemental analysis (EDX) (Figure 4G) showed a high content of carbon, nitrogen, and oxygen elements corresponding to chitosan, PNIPAm, and curcumin in the nanogel structure, Moreover, the presence of sulfur and gold in the elemental analysis verified the synthesis of thiolated chitosan and existence of AuNP in the structure of AuNP@Ng/Cur (Sidhu et al., 2019).

### Curcumin Loading and Encapsulation Efficiency in AuNP@Ng

Curcumin is classified as a Biopharmaceutics Classification System (BCS) Class IV substance and thus, exhibits both poor solubility and permeability (Wang et al., 2017). Consequently, low bioavailability, low solubility in water, and rapid metabolism of curcumin lead to profound problems in inducing an anti-cancer effect. Here, using a plasmonic nanogel as a drug delivery system could protect curcumin from rapid metabolism inside the body and could circumvent any solubility and permeability issues, since these would be determined by the carrier. The higher the drug loading and EE% of a carrier, along with low premature drug loss would overall result in a more significant impact. It has been reported that polymeric Ng have promising potential as drug delivery carriers in terms of drug loading capacity, biocompatibility, and thermal responsivity (Liechty and Peppas, 2012). It is expected that the interaction between nanogel and curcumin is hydrophobic due to the interaction between curcumin and PNIPAM (Asghar et al., 2017). The curcumin loading capacity and EE% of nanogel were measured spectrophotometrically and calculated before and after conjugation with AuNP with two different concentrations of curcumin (0.25 and 0.5 mg/ml). The highest DL% (17%) and EE% (92%) were found with AuNP@Ng formulation at a concentration of 0.5 mg/ml curcumin (Table 3). The higher EE% and DL% of AuNP@Ng compared to nanogel is probably related to the ability of curcumin to conjugate to AuNP (Luckanagul et al., 2018). Moreover, it has been observed that the conjugation efficiency of curcumin to AuNP increases with respect to time (Mahalunkar et al., 2019). In this study, it was thought that the 24-h mixing time after adding curcumin to the formulation also increased the conjugation of curcumin to gold nanoparticles. Ng, on the other hand, have the ability to trap drugs due to the presence of an internal network structure(Ghorbani et al., 2016; Vashist et al., 2018). Therefore, it was thought that higher loading capacity was obtained in the AuNPs@Ng formulation with the combination of these two methods. Furthermore, as mentioned, UV-VIS absorption of AuNP@Ng/Cur confirmed the encapsulation of curcumin by the nanogel, as it exhibited a peak at 450 nm wavelength in addition to the AuNP peak, which indicates the presence of curcumin (Alam et al., 2012).

**Table 3:** DD% and EE% of nanogel before and after conjugation with AuNP.

**Nanoparticle**	**Concentration of curcumin (mg/ml)**	**DL (%) ± SD**	**EE (%) ± SD**
Ng	0.25	4.7 ± 0.57%	69.03 ± 1.39%
Ng	0.5	8.04 ± 0.25%	90.03 ± 1.01%
AuNP @Ng	0.25	9.2 ± 0.43%	84 ± 1.62%
AuNP @Ng	0.5	17.01 ± 0.33%	92 ± 0.98%

### Thermo-pH-Responsive Curcumin Release From AuNP@Ng

**Figure 5:**
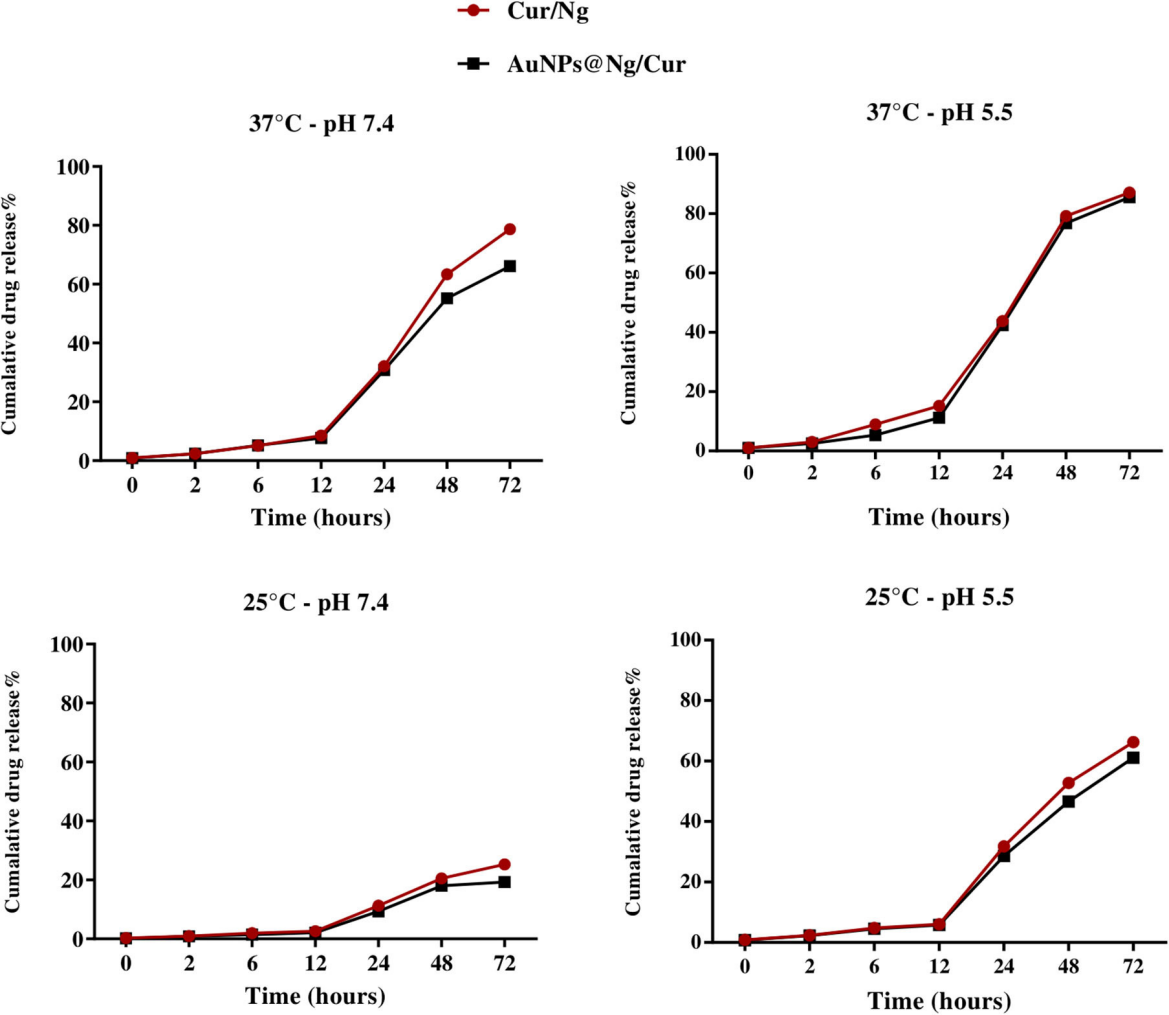
*In vitro* curcumin release at different pH (5.5 and 7.4) and different temperatures (25 and 37°C) from Cur/Ng and AuNP @Ng/Cur (*n* = 3).

One of the major benefits of nanogels is its thermo-responsive property that enables controlled drug release in the cellular environment with an on-off trigger mechanism. In order to evaluate the thermo-pH sensitivity of nanogel, the *in vitro* release of curcumin from Ng and AuNP@Ng was carried out at two different temperatures of 25 and 37°C under pH conditions of 5.5 and 7.4 in a dissolution medium containing tween 20 to provide sink conditions in the release environment (Shahani and Panyam, 2011; Ching et al., 2019). According to statistical analysis, there are significant differences in drug release between the 4 different conditions of 37°C pH 5.5, 37°C pH 7.4, 25°C pH 5.5, and 25°C pH 7.4 for nanogel and AuNP@Ng (^****^P < 0.0001). The release profiles of curcumin from the Ng and AuNP@Ng shown in Figure 5, demonstrated no burst release, which indicated that curcumin was not adsorbed to the nanogel surface and was entirely encapsulated in the nanogel structure. Higher curcumin release was observed at acidic pH compared to the release at physiological pH. Drug release reached equilibrium at 80% after 72 h for both nanogel and AuNP@Ng formulations at 37°C and acidic pH. Only 20% of the drug was released from AuNP@Ng within 72 h at 25°C and pH 7.4. The low critical solution temperature (LCST) properties of PNIPAM cause an extraordinary shrinkage, leading to curcumin release at temperatures higher than LCST; whereby the drug release only depends on free diffusion resulting in lower drug release. The significant size transition measured by DLS confirmed the gradual shrinkage of nanogel in temperatures above LCST (Figure 3B; Kim et al., 2019). Thus, using chitosan in this formulation not only improved the LCST properties of nanogel by increasing it to the body temperature, but also, the pK_a_ value of linear chitosan chains make the nanogel pH-responsive and this leads to the rupture of the Ng under acidic conditions, followed by curcumin release (Pujana et al., 2012). Since the tumor cells have an acidic environment, curcumin-loaded Ng appear to be advantageous to deliver the encapsulated drug to tumor cells (Swietach et al., 2014). Hyperthermia generated by Au nanoparticles under NIR irradiation can stimulate drug release from particles after cellular uptake and could effectively reverse drug resistance of tumor cells, which highly enhanced the killing effects of chemotherapeutics and promoted cell apoptosis (Li et al., 2019; Gao et al., 2020).

### Cellular Viability of AuNP@Ng/Cur

**Figure 6:**
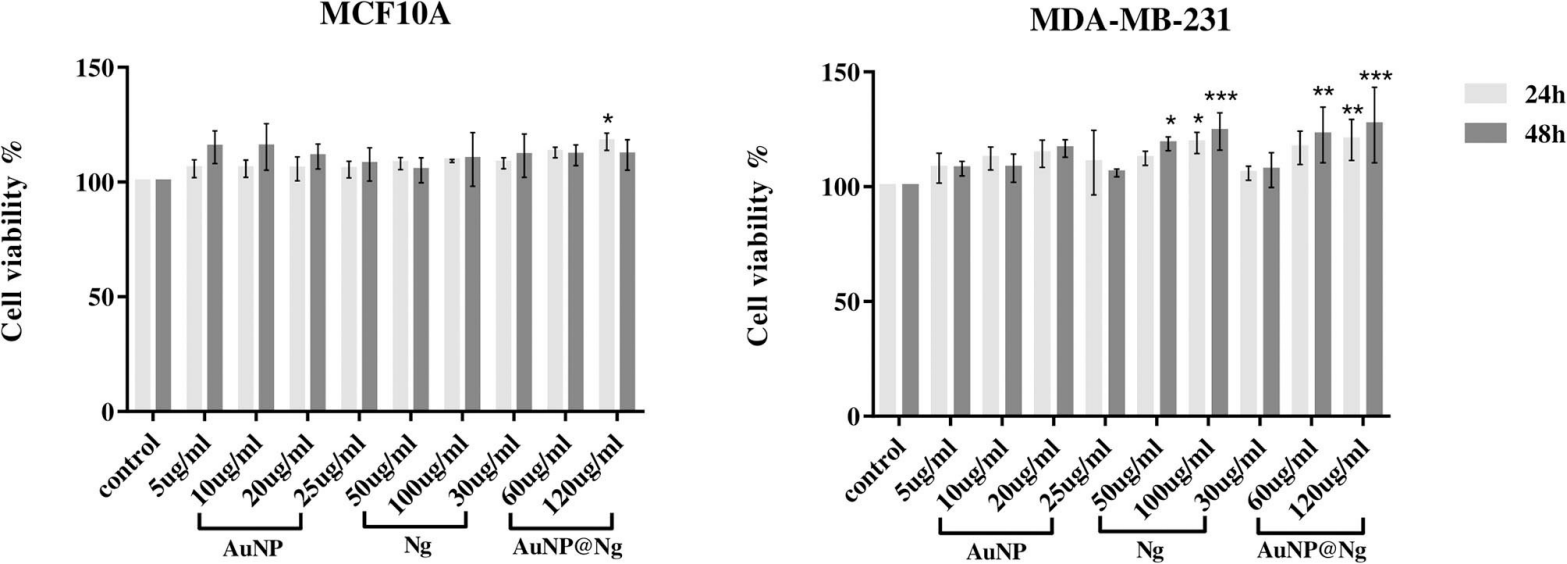
Cellular viability of MDA-MB-231 and MCF-10A cells treated with Ng (25, 50, and 100 μg/ml), AuNP (5, 10, and 20 μg/ml), and AuNP@Ng (30, 60, and 120 μg/ml) nanoparticles after 24 and 48 h treatment. Data are presented as the mean ± standard deviation (SD) (*n* = 4). Two-way ANOVA followed by Tukey's multiple comparisons test was performed to investigate the significant difference (***p < 0.0001-0.001; **p < 0.001-0.01; *p < 0.01).

Cell viability of the nanoparticles was investigated in terms of the effect on the cell proliferation by Alamar Blue assay. MDA-MB-231 and MCF10A cells were treated with different concentrations of AuNP@Ng and the corresponding concentrations of individual AuNP and Ng for 24 and 48 h, as shown in Figure 6, The performed statistical analysis for both MDA-MB-231 and MCF 10A cells treated with nanoparticles using two-way ANOVA revealed no significant difference in cell viability between two time points (*p* > 0.05). According to Tukey's multiple comparisons test, there is significant differences in cell viability of MCF10A cells in sample treated with AuNP@Ng 120 μg/ml in 24 h in comparison with control sample (^*^p < 0.01). Same statistical analysis for MDA-MB-231 cells, showed significant difference in cell viability for samples treated with nanogel 100 μg/ml and AuNP@Ng 120 μg/ml in 24 h in comparison with control sample (respectively, ^*^p < 0.01 and ^**^p < 0.001). Moreover, there are significant differences in increasing the cell viability of MDA-MB-231 cells for samples treated with Ng 50 μg/ml, Ng 100 μg/ml, 60 μg/ml AuNP@Ng, and 120 μg/ml AuNP@Ng after 48 h in comparison with control sample (respectively, ^*^p < 0.01; ^**^p < 0.01; ^**^p < 0.01; ^***^p < 0.001). These findings show that nanoparticle treated MDA-MB-231 and MCF10A cells exhibited slightly higher viability in comparison to control cells (100%). These results suggest that AuNP may have a promoting effect on cell proliferation of MDA-MB-231 and MCF10A cells at low concentrations (Gao et al., 2020). Thus, these results indicated that AuNP@Ng nanocarriers are safe for *in vitro* applications where the concentration is below 120 μg/ml.

### Intracellular Uptake of AuNP@Ng and AuNP@Ng/Cur Nanoparticles

**Figure 7:**
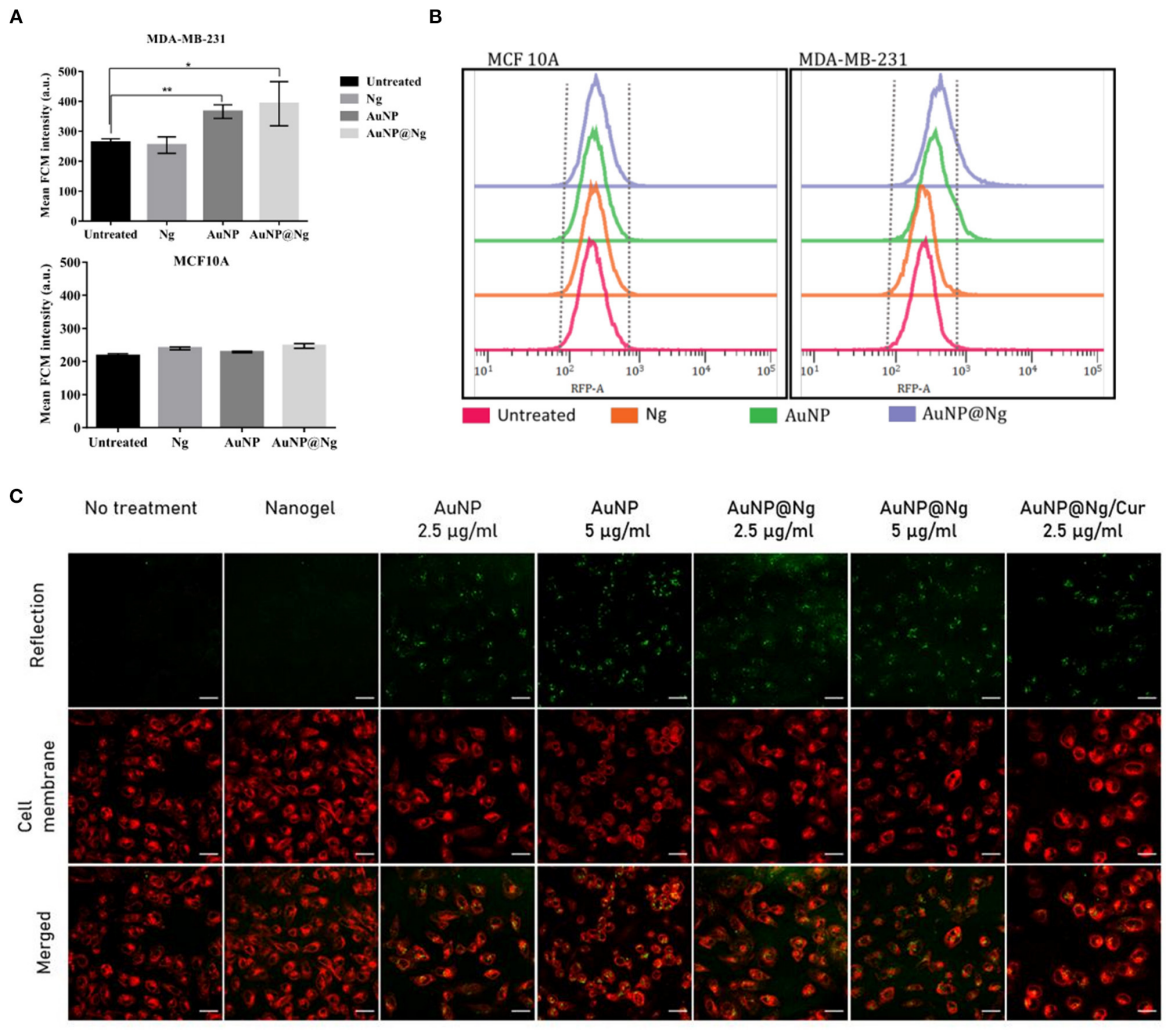
Cellular uptake study of nanoparticles Quantification of cellular uptake by flow cytometry after 24-h incubation of Ng, AuNP, and AuNP@Ng with MDA-MB-231 breast cancer cell line and non-tumorigenic MCF 10A cell line. Significant differences were observed in cellular uptake of nanoparticles in the statistical analysis using the two-tailed Student *t*-test (**p < 0.0001–0.001; *p < 0.001–0.01) by MDA-MB-231 cells when treated with AuNP and AuNP@Ng compared to untreated samples **(A)**. AuNP intensity histograms detected by RFP filter representing Ng, AuNP, and AuNP@Ng cellular internalization in MDA-MB-231 and MCF 10A cells **(B)**. Confocal microscopy images of MDA-MB-231 cells incubated with AuNP (2.5 and 5 μg/ml), AuNP@Ng (2.5 and 5 μg/ml), and AuNP@Ng/Cur for 24 h. AuNP are acquired with reflection imaging (green), the cell membrane was stained with carbocyanine dye DiI (red). The scale bar is 30 μm **(C)**.

Following the cellular viability, cellular internalization quantification of the AuNP@Ng nanocarriers was investigated by employing the AuNP detection by flow cytometry. The incubation duration was fixed to 24 h as most of the doubling time of mammalian cell lines are longer than 24 h, and thus cell proliferation effects such as dilution of intracellular nanoparticles due to cell cycling would be eliminated (Shin et al., 2020). Traditionally, AuNPs are detected by the side scattering mode with 488 nm laser illumination (SSC channel). However, this mode of action was found to be not sensitive enough for our application, presumably due to detection range not being close to the SPR peak of AuNPs, and the difference being insignificant due to the small AuNPs particle size and low particle concentration. The other proposed method(Wu et al., 2019) was employing the SPR phenomena of AuNP which gives peak in the 510-550 nm range, and the aggregation of AuNP inside the cells causes a red shift in the SPR peak(Liu et al., 2017). Therefore, this mode of action is more sensitive to detect the scattered light in a flow cytometer. When the excitation laser 561 nm and corresponding 582/15 band pass filter (RFP filter) was used, MDA-MB-231 cells that internalized AuNP containing nanoparticles showed significant difference in flow cytometry intensity (FCM) (respectively, ^**^p < 0.001 and ^*^p < 0.01) (Figure 7A). Both AuNP and AuNP@Ng showed similar results, whereas the signal intensity of Ng internalized cells was similar to the untreated cells suggesting that the signal is specific to AuNPs, and nanogel-AuNP conjugation does not alter the detection of AuNPs. However, no significant difference was observed for MCF 10A non-tumorigenic cell line (*p* > 0.05). This could be attributed to the slower metabolism of non-tumorigenic cell lines compared with cancer cells (Zancan et al., 2010), resulting with the internalized nanoparticle concentration being below detection range. Additionally, Figure 7B, demonstrated that following AuNP or AuNP@Ng nanoparticle treatment, histograms on RFP channel shifted to larger intensities, whereas Ng nanoparticle treatment showed no histogram shift, suggesting the FCM intensity detection was specific to AuNPs. To verify the presence and location of the nanoparticles inside the cells, AuNP, AuNP@Ng treated cells were imaged with confocal microscopy after 24 h incubation. AuNP were detected by using reflection imaging due to its extraordinary efficiency of light absorption and emission. The cell membrane was stained with carbocyanine dye DiI in order to explore the cellular internalization pattern of nanoparticles, as DiI dye stains entire lipid components of the cell (Jensen and Berg, 2016). The scattered light signals of AuNP were bright enough to be distinguished from cellular autofluorescence (Figure 7C) when the reflection imaging settings were optimized for image acquisition. Nanoparticles were efficiently internalized by MDA-MB-231 cells and localized in the cytoplasm as aggregates suggesting their endosomal entrapment (Kim et al., 2015). Acquired images suggested dose-dependent internalization with the observations of higher concentration of AuNP and AuNP@Ng, resulting in denser aggregations inside the cells. Recently, it was reported that gelatin/protein Ng serve as reducing and stabilizing agents for the AuNP by allowing for nucleation in a gel network that exhibits colloidal stability (Chen et al., 2019). Our results showed similar results in confocal microscopy, AuNP@Ng showed more dispersed aggregates compared with densely packed AuNP.

### Curcumin Delivery and PTT Efficiency Evaluations of AuNP, AuNP@Ng, and AuNP@Ng/Cur Against MDA-MB-231 Cells

**Figure 8:**
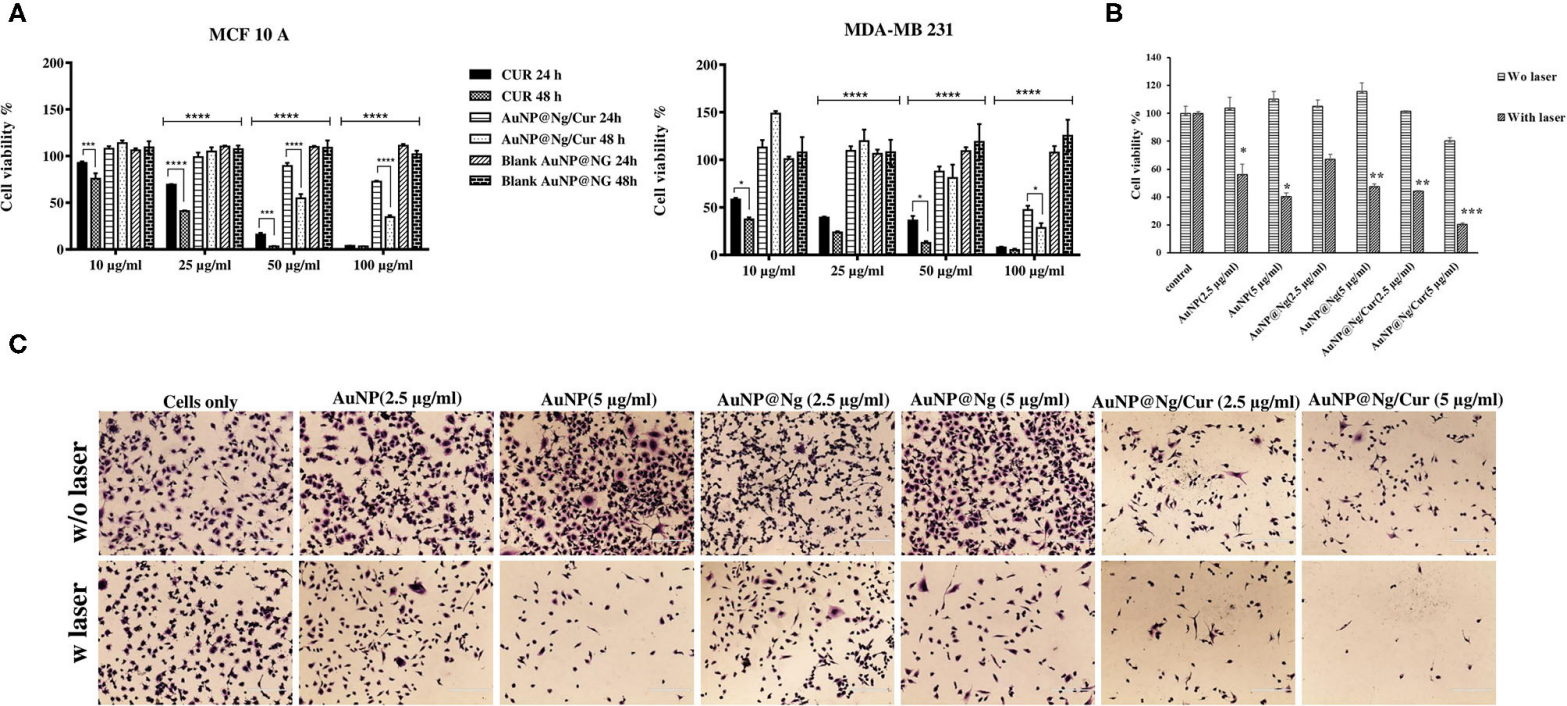
Cytotoxicity of free curcumin and AuNP@Ng/Cur incubated with MDA-MB-231 and MCF-10A cells for 24 and 48 h. Data are presented as the mean ± standard deviation (SD) Two-way ANOVA followed by Tukey's multiple comparisons test was performed to investigate the significant difference (****p < 0.0001; ***p < 0.0001–0.001; **p < 0.001–0.01; *p < 0.01) **(A)**. Cell viability of MDA-MB-231 cells treated with AuNP, AuNP@Ng, and AuNP@Ng/Cur after exposure to NIR laser **(B)**. Crystal violates -stained MDA-MB-231 cells treated with AuNP (2.5–5 μg/ml), AuNP@Ng (2.5–5 μg/ml), and AuNP@Ng/Cur (2.5–5 μg/ml) after laser exposure for 10 min (Scale bar is 200 μm) **(C)**.

The potential anticancer ability of AuNP@Ng/Cur was evaluated by using MDA-MB-231 and MCF10A cells directly comparing its time and dose-dependent efficacy to free curcumin performed by Alamar Blue cell proliferation assay. According to obtained statistical analysis using two-way ANOVA followed by Tukey's multiple comparisons test for MDA-MB-231 and MCF10A cells, there are significant dose dependant decrease in cell viability for both samples treated with curcumin and AuNP@Ng/Cur in comparison with samples treated with 10 μg/ml concentration of curcumin for both time points Figure 8A (*p* < 0.05). Therefore, these findings demonstrate the inhibition of the proliferation of cells at the tested concentrations, which increase by increasing the concentration of the treatments (Zancan et al., 2010). For both MDA-MB-231 and MCF10A cells there are time dependent significant differences in cell viability for samples treated with 100 μg/ml (*p* < 0.05). AuNP@NG/Cur nanoparticles had lower proliferation inhibition efficiency when compared with free curcumin. These results suggested nanogels having stimuli-responsive and sustained drug release profile and requiring a longer period for drug release in comparison with the immediate access of free curcumin for cells (Sultana et al., 2013). According to Figure 5, ~75% of the drug was released after 48 h. This sustained-release pattern might be the reason for the lower inhibition in cell proliferation in the sample treated with AuNP@Ng/Cur.

In addition to curcumin delivery, the photothermal therapeutic effect of AuNP@Ng/Cur against MDA-MB-231 cells was evaluated by applying NIR irradiation after incubating cells with AuNP@Ng/Cur containing 2.5 and 5 μg/ml AuNP. After 24 h of incubation, cells were exposed to NIR irradiation for 10 min. AuNP represent LPSR, which has the ability in absorbing NIR light and converting it to heat. This heat generation causes the shrinkage of nanogel and curcumin release by triggering the thermoresponsive properties of synthesized AuNP@Ng/Cur nanoparticles (Vines et al., 2019). The Alamar blue assay and crystal violet staining after PTT indicated that AuNP, AuNP@Ng, and AuNP@Ng/Cur could significantly decrease the cell viability of MDA-MB-231 cells compared to the control cells with no NIR laser exposure, which were viable after irradiation NIR laser (*p* < 0.05). These results clearly showed the significant photothermal ability of the synthesized nanoparticles against MDA-MB-231 cells when treated with AuNP@Ng/Cur, in addition to increased concentration of AuNP in AuNP@Ng/Cur and AuNP@Ng being able to remarkably increase the photothermal efficiency of AuNP@Ng/Cur (Figures 8B,C) (*p* < 0.05). Although drug loaded AuNP@Ng nanoparticles showed less anticancer efficiency, our novel dual therapy nanoparticle system constitutes a potential delivery approach with overall advantages of Ng being cytocompatible and containing AuNP for PTT and enhancing the solubility of poorly water-soluble drugs.

## Conclusion

Plasmonic nanogels are of interest to a range of medical fields, including hydrophobic drug delivery and bioimaging, due to their high biocompatibility, biodegradability, and stimuli responsivity. To date, various nanogels have been synthesized using different synthetic methods. Although there is considerable progress in the success of nanogels, in practice they suffer from multiple dilemmas such as high toxicity, burst release, and poor diagnostic sensitivity. Therefore, in this study, biocompatible, stimuli-responsive plasmonic nanogel was successfully synthesized in the presence of an MBA crosslinker with free radical surfactant-free emulsion polymerization method by grafting PNIPAM to thiolated chitosan. Having isopropyl hydrophobic groups rendered the AuNP@Ng an appropriate nanocarrier for a wide range of anticancer drugs with low solubility. Sustained curcumin release pattern by AuNP@Ng was achieved for 72 h. Our developed AuNP@Ng/Cur nanoparticles conveniently overcame the cellular barrier and entered the MDA-MB-231 cells, which was observed through confocal microscopy. The pH-thermoresponsive plasmonic nanogel was found to have efficient toxicity against MDA-MB-231 cells, which increased with the combination of photothermal therapy. Thus, it can be suggested that AuNP@Ng could be introduced as a biocompatible multifunctional nanocarrier for the dual delivery of curcumin and photothermal therapy.

## Data Availability Statement

The original contributions presented in the study are included in the article/supplementary materials, further inquiries can be directed to the corresponding author/s.

## Author Contributions

FH: investigation, conceptualization, methodology, data analysis, writing—original draft, and writing—review and editing. EÖ: investigation, methodology, data analysis and writing—original draft, and writing—review and editing. BK: methodology and writing—review and editing. SR: methodology. MS: supervision, investigation, validation, and funding acquisition and editing. JR: supervision, validation, funding acquisition, and writing—review and editing. All authors contributed to the article and approved the submitted version.

## Conflict of Interest

The authors declare that the research was conducted in the absence of any commercial or financial relationships that could be construed as a potential conflict of interest.
